# Phenotypic Characteristics of the Tumour Microenvironment in Primary and Secondary Hepatocellular Carcinoma

**DOI:** 10.3390/cancers13092137

**Published:** 2021-04-29

**Authors:** Petros Fessas, Paolo Spina, Renzo L. Boldorini, Mario Pirisi, Rosalba Minisini, Francesco A. Mauri, Fraser Simpson, Paola Olivieri, Alessandra Gennari, Ching Ngar Wong, Abdul Siddique, Robert D. Goldin, Ayse U. Akarca, Teresa Marafioti, David J. Pinato

**Affiliations:** 1 Department of Surgery & Cancer, Imperial College London, Hammersmith Hospital, Du Cane Road, London W120 HS, UK; pfessas@ic.ac.uk (P.F.); f.mauri@imperial.ac.uk (F.A.M.); wongchingngar@gmail.com (C.N.W.); abdul.siddique12@imperial.ac.uk (A.S.); 2 Cantonal Institute of Pathology, Via in Selva 24, 6601 Locarno, Switzerland; paolospina86@yahoo.it; 3 Department of Health Sciences, Universitá degli Studi del Piemonte Orientale “A. Avogadro”, Via Solaroli 17, 13100 Novara, Italy; renzo.boldorini@med.uniupo.it; 4 Department of Translational Medicine, Università degli Studi del Piemonte Orientale, 13100 Novara, Italy; mario.pirisi@med.uniupo.it (M.P.); rosalba.minisini@med.uniupo.it (R.M.); alessandra.gennari@med.uniupo.it (A.G.); 5 Department of Genetics, Evolution and Environment & Cell and Developmental Biology, University College London, London WC1E 6BT, UK; f.simpson@ucl.ac.uk (F.S.); p.olivieri@ucl.ac.uk (P.O.); 6 Centre for Pathology, Imperial College London, London SW7 2AZ, UK; r.goldin@imperial.ac.uk; 7 Department of Histopathology, University College London Hospital, London SW7 2AZ, UK; a.akarca@ucl.ac.uk (A.U.A.); t.marafioti@ucl.ac.uk (T.M.); 8 Clinical Senior Lecturer and Consultant in Medical Oncology, Imperial College London Hammersmith Campus, Du Cane Road, London W12 0HS, UK

**Keywords:** hepatocellular carcinoma, intra-tumoural heterogeneity, tumour microenvironment

## Abstract

**Simple Summary:**

How liver cancer changes as it progresses from where it initially arises, to metastatic sites distant from the liver, is unclear. We obtained a unique set of paired samples from patients, both from the original site and from metastases. We compared the mutation burden, transcriptional profile, and immune cell infiltrate between primary and secondary samples. We found that liver cancer metastases retain the ability to exclude the immune system from the tumour core as they spread.

**Abstract:**

(1) Background: The intra-tumoural heterogeneity (ITH) of hepatocellular carcinoma (HCC) and its microenvironment (TME) across primary and secondary disease is poorly characterised. (2) Methods: Intra-tumoural (IT) and peri-tumoural (PT) staining of matched primary and secondary samples was conducted to evaluate the distribution of CD4+/FOXP3+ and CD8+/PD1+ T-cells. Samples underwent PD-L1/2 immunostaining, tumour mutational burden (TMB) evaluation, and high-resolution T-cell receptor (TCR) sequencing to derive T-cell clonality and targeted transcriptomics. (3) Results: We analysed 24 samples from matched primary (*n* = 11) and secondary (*n* = 13; 5 synchronous, 6 metachronous) deposits, 11 being extrahepatic (84.6%). IT CD8+ density was lower than PT in both primary (*p* = 0.005) and secondary deposits (*p* = 0.01), consistent with immune exclusion. PD-L1+ tumours displayed higher IT and PT CD8+/PD1+ cell density compared to PD-L1- (*p* < 0.05), and primary IT infiltrate was enriched in CD4+/FOXP3+ cells, compared to PT regions (*p* = 0.004). TCR-sequencing demonstrated enrichment of the top T-cell clonotype in secondary versus primary HCC (*p* = 0.02), without differences in overall productive clonality (*p* = 0.35). TMB was similar across primary versus secondary HCC (*p* = 0.95). While directed gene set analysis demonstrated the uniformity of transcriptional signatures of individual immune cell types, secondary deposits demonstrated higher *COLEC12* (*p* = 0.004), *CCL26* (*p* = 0.02), *CD1E* (*p* = 0.02) and *CD36* (*p* = 0.03) expression with downregulation of *CXCL1* (*p* = 0.03), suggesting differential regulation of innate immunity. (4) Conclusion: Immune exclusion is a defining feature of the HCC TME. Despite evidence of homogeneity in somatic TMB, secondary HCC is characterised by the expansion of a distinct T-cell clonotype and differential regulation of innate immune pathways.

## 1. Introduction

The contemporary management of advanced hepatocellular (HCC) recognises tyrosine kinase inhibitors (TKI) and immune checkpoint inhibitors (ICI) as the mainstay of treatment [1]. The dual inhibition of programmed cell death ligand 1 (PD-L1) and vascular endothelial growth factor (VEGF), with atezolizumab and bevacizumab, has recently emerged as a more efficacious option than sorafenib [1], although long-term survival data from this combination are unknown. Unlike other oncological diagnoses, where highly prevalent truncal genomic abnormalities indicate responsiveness to molecular therapies, there are no biomarkers to guide treatment selection in HCC. None of the molecular therapies approved for HCC work through selective inhibition of a single molecular abnormality, and tissue-based predictors of benefit from immune checkpoint inhibitors (ICI), such as PD-L1 expression and tumour mutational burden (TMB), lack predictive ability and are characterised by pre-analytic heterogeneity [2].

Intra-tumour heterogeneity (ITH) is a recognised feature of evolving malignancies, where the diverse coexisting neoplastic subclones accumulate serial genetic and epigenetic modifications in space and time, with ultimate implications in their differential sensitivity to treatment [3]. The significance of ITH as a predisposing factor in poor clinical outcomes has been demonstrated across cancer types [4], and several studies have demonstrated ITH in HCC [5], where greater heterogeneity is associated with adverse outcomes [6].

## 2. Materials and Methods

### 2.1. Patient Samples

From a multicentre repository of archival HCC specimens, we retrieved specimens of paired primary HCC lesions and secondary deposits. Formalin-fixed paraffin-embedded (FFPE) tissue blocks were retrieved. Diagnosis of HCC and the distribution of secondary sites were confirmed histologically on newly cut Haematoxylin and Eosin (H&E) sections by a consultant liver pathologist (RG).

### 2.2. Immunohistochemistry (IHC)

Two-micron tissue sections underwent single-marker immunostaining for Programmed Cell-death ligands 1 (PD-L1) and 2 (PD-L2), using antibody clones E1L3N (Cell Signalling Cat. Nr. 13684, dilution 1:100) and D7U8C (Cell Signalling Cat. Nr. 82723, dilution 1:100), respectively, on a Leica Bond RX stainer (Leica, Buffalo, IL, USA). Multiplex immunostaining for CD4 (Spring Biosciences, Pleasanton, CA, USA clone SP35), CD8 (clone SP239), FOXP3 (BD Biosciences, San Jose, CA, USA, clone 346/E7) and PD-1 (Novus Biologicals, Centennial, CO, USA, clone NAT 105/E3) followed a pre-optimised protocol [7]. Individual counts of CD4+, CD8+, CD4+/FOXP3+ and CD8+/PD-1+ co-immunopositive cells were performed in tissue photomicrographs, assessed at 450× magnification across intra-tumoural (IT) and peri-tumoural (PT) areas and reported as cellular density per mm^2^ of tissue. PD-L1/2 expression in tumour cells was presented using a semi-quantitative score (H-score, range 0–300), derived from multiplying the percentage of positive cells (1% cut-off) by chromogenic intensity (ranked from 0 to 3). Immunopositivity was scored categorically, using a 1% cut-off value, as routinely employed in clinical trials of ICI.

### 2.3. DNA and RNA Purification

The tissue specimens were quality controlled by a consultant histopathologist (FAM) on H&E sections to identify target areas containing >20% of viable tumour, prior to molecular profiling. DNA and RNA were purified from 10 µM-thick FFPE sections, using the AllPrep DNA/RNA FFPE Kit (Qiagen, Manchester, UK, Cat. 80234). RNA and DNA quantification and quality control were performed on an ND2000 Nanodrop spectrophotometer (Thermo Fisher Scientific, Loughborough, UK). DNA samples were further measured using a QubitTM Flex Fluorometer 2.0 (Thermo Fisher Scientific).

### 2.4. Targeted Next Generation Sequencing (tNGS)

tNGS of tumoural DNA was performed on an Ion PGM sequencer (Thermo Fisher Scientific) and the Ion AmpliSeq Cancer Hotspot Panel v2, which amplified 207 amplicons covering 2800 mutations from 50 loci (0.226 Mbp coverage, Thermo Fisher Scientific), which were all included in the core COSMIC database due to their relevance to cancer, based on the scientific literature, after expert curation [8]. Following histological assessment with H&E-stained sections by a pathologist (FAM) to confirm tumour cell contents (tumour purity), the tumour areas of the FFPE sections were macro-dissected. The minimal tumour cellularity for the NGS test was 20%. The Ion Reporter suite (Life Technologies, Waltham, MA, USA) was used to filter polymorphic variants. Alongside the individual variant calling, we expressed the sample TMB as number of mutations/Mb by counting any non-synonymous mutation with a variant allelic fraction >0.05. The cut-off to define TMB-high samples was adjusted to >2 Mut/Mb to reflect the coverage of the tNGS platform utilised.

### 2.5. NanoString Immune Profiling

Targeted transcriptomic profiling was performed on 200 ng of the total RNA extracted, following H&E-guided microdissection of target tumour tissue, using the NanoString PanCancer Immune panel on an nCounter^®^ Analysis System (NanoString Technologies, Seattle, WA, USA, Supplementary Methods), and analysed using the nSolver Analysis Software (NanoString Technologies, Seattle, WA, USA).

### 2.6. High-Resolution T-Cell Receptor Sequencing

The immunoSEQ Assay (Adaptive Biotechnologies, Seattle, WA, USA) was used to sequence the CDR3 regions of human TCR beta chains from purified tissue DNA samples, as described previously [9]. For each sample, TCRB CDR3 regions were amplified and sequenced up to 2 ug of genomic DNA. TCRB CDR3 regions were identified within the sequencing reads, in accordance with the immunogenetics definition [10]. T-cell density was calculated by normalising TCR template counts to the total amount of DNA that was usable for TCR sequencing. The amount of usable DNA was determined by polymerase chain reaction (PCR) amplification and the sequencing of housekeeping genes. Clonality was computed on productive rearrangements and defined as 1-Pielou’s evenness [11].

### 2.7. Statistical Analysis

Descriptive statistics are presented as medians or means. Comparisons of proportions were performed using Pearson’s Chi-Square or Fisher’s exact tests and comparisons of medians were evaluated by Mann–Whitney U tests. Pearson’s or Spearman’s correlation coefficient tests were used to investigate correlations between clinicopathological variables. Differential expression of specific genes in RNA expression experiments was determined using the false discovery rate (FDR) method of Benjamini and Hochberg, with a pre-defined q-value of 5%. All statistical analyses were performed using SPSS, version 26.0 (IBM Inc., Chicago, IL, USA) and GraphPad Prism (GraphPad software Inc., La Jolla, CA, USA). All estimates were reported with 95% confidence intervals (CI) and a two-tailed level of significance of *p* ≤ 0.05.

## 3. Results

### 3.1. Clinicopathologic Characteristics

After careful review of electronic pathology records of two tertiary academic centres specialised in the care of patients with HCC (Novara, Italy and Imperial College London, United Kingdom), we analysed 24 individual deposits from primary (*n* = 11) and secondary HCC (*n* = 13), derived from 11 patients with histologic diagnosis of HCC between 1996 and 2013. Secondary deposits were mostly extrahepatic (*n* = 11, 84.6%) and were synchronous (identified at the time of the diagnosis of the primary lesion) in five (45.5%) and metachronous in six cases (54.5%) (identified after the diagnosis of the primary lesion). The median time to relapse in metachronous tumours was 2 years (range 1–7). Clinicopathologic features and distribution of secondary sites are presented in Table 1.

### 3.2. Functional Characterisation of the T-Cell Infiltrate across Primary and Secondary HCC

**Figure 1:**
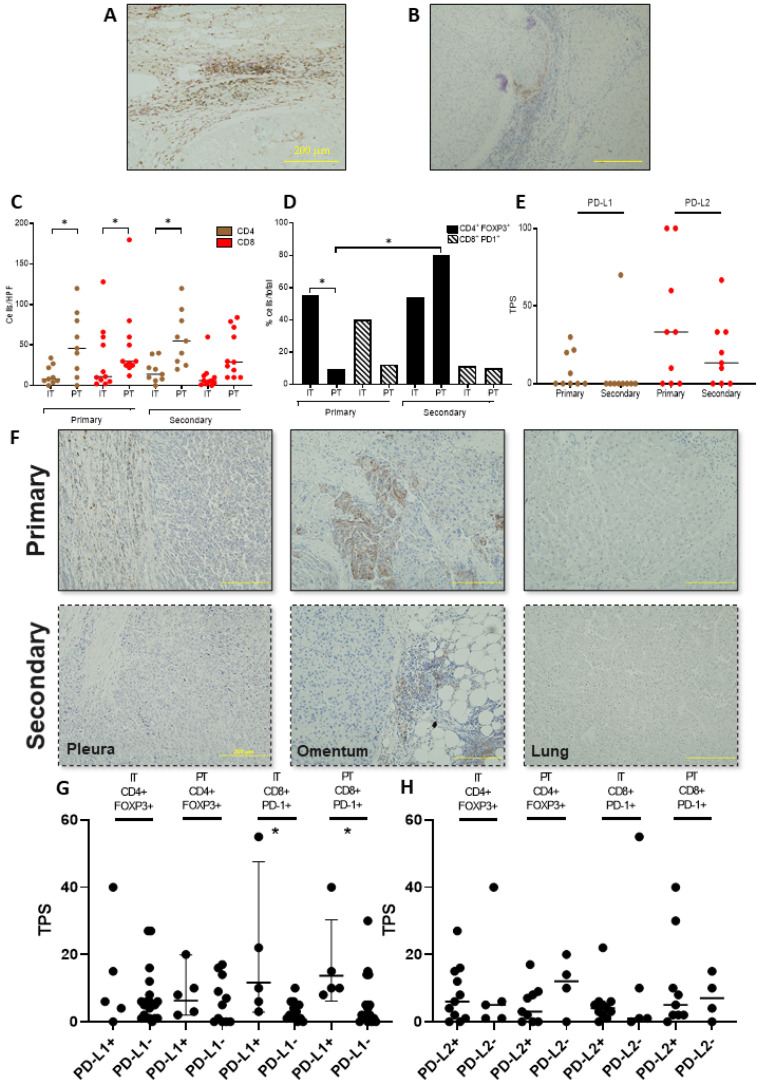
Immunohistochemical characterisation of the immune cell infiltrate in primary and secondary HCC. Representative sections illustrating the characteristics of the T-cell infiltrate in a primary HCC sample. A red “T” highlights the tumoural area (magnification 200×). T-cell phenotypic characteristics are evaluated by multiplex immunohistochemistry for CD4 (brown chromogen), FOXP3 (green chromogen), CD8+ (red chromogen), PD-1 (blue chromogen), and CD8+/PD1 co-expression (purple chromogen) and show evidence of restriction of T-cell localization in non-tumoural areas, adjacent to the tumour margin. (**A**). Focal representative section of the same sample, depicted in (**A**) showing expression of PD-L1 at the peripheral budding margin of the primary HCC deposit, where evidence of a PD-L1 immunopositive infiltrate can be seen at the margin of the lesion (**B**). Immunohistochemistry estimates of the density of CD4+ and CD8+ cells (**C**) and the CD4+FOXP3+ and CD8+PD-1+ cells (cells/mm^2^) (**D**). Tumour proportion scores (TPS) of PD-L1 and PD-L2 expression in primary and secondary samples (**E**). Representative sections highlighting the heterogeneity of PD-L1 expression across primary and secondary HCC sites. In the top row, three examples of primary HCC deposits testing positive (straight line) or negative (dashed line) for PD-L1, presented in association with matched metastatic deposits in the bottom row. (**F**) Cell density (cells/mm^2^) of CD4+FOXP3+ and CD8+PD-1+ cells analysed according to PD-L1 (**G**) and PD-L2 (**H**) expression. * *p* < 0.05.

Firstly, we evaluated the T-cell infiltrate on intra-tumoural (IT) and peri-tumoural (PT) areas of archival samples, using immunostaining (Figure 1A,B). Whilst the total CD4+ and CD8+ cell counts were not differentially distributed when comparing IT and PT of primary and secondary sites, we found evidence of significant immune exclusion, documented by a negative IT/PT cell density gradient in both primary (CD4+ IT/PT 13.0 versus 51.9 cells/mm^2^, *p* = 0.01; CD8+ IT/PT 17.7 versus 58.2 cells/mm^2^, *p* = 0.005) and secondary samples (CD8+ IT/PT 32.5 versus 49.3 cells/mm^2^, *p* = 0.01), with the exception of CD4+ cells in secondary samples, which were equally distributed across IT and PT areas (IT/PT 10.9 versus 38.8 cells/mm^2^, *p* = 0.38, Figure 1C). We found an enrichment of CD4+/FOXP3+ over total CD4+ T-cells in IT (52.7%) over PT regions (13.0%, *p* = 0.004) in primary but not in secondary samples (46.9 versus 66.2%, *p* = 0.18), whereas the proportion of CD8+/PD-1+ cells was similar in IT and PT regions in both primary (48.3% versus 21.7%, *p* = 0.08) and secondary HCC (24.8% versus 23.8%, *p* = 0.92) (Figure 1D).

### 3.3. PD-L1/2 Expression Influences the Tumour Microenvironment in Primary and Secondary HCC

We found significant heterogeneity in the tumoural expression of PD ligands across primary and secondary samples. Of the three PD-L1-positive primaries, only one had a matched PD-L1-positive secondary, whereas all four PD-L1-negative primaries had matched PD-L1-negative secondary samples. Of the six PD-L2-positive primaries, five had concordant PD-L2-positive secondary samples, whereas all PD-L2-negative primaries had matched PD-L2-negative secondaries (Figure 1E,F). We formally evaluated the concordance of immunolabelling status across primary and secondary HCC, using Cohen’s Kappa, demonstrating evidence of greater discordance for PD-L1 (Cohen’s Kappa = 0.25), compared to that for PD-L2 expression across sample groups (Cohen’s Kappa = 0.71). We further examined whether PD ligand expression on tumour cells correlated with the density of immune-exhausted PD-1+ cells in our samples. PD-L1 positivity was associated with higher CD8+PD-1+ cell density in both primary and secondary samples, both in PT regions (5.9 in PD-L1- versus 16.6 cells/mm^2^ PD-L1+, *p* = 0.04) and IT regions (3.3 in PD-L1- and 19.2 in PD-L1+, *p* = 0.01). This was not the case for PD-L2, where tumoural expression of the ligand was not associated with differences in the phenotypic characteristics of the T-cell infiltrate (*p* = 0.63, Figure 1G,H).

### 3.4. Deep Sequencing of the T-Cell Receptor T-Cell Infiltrate across Primary and Secondary HCC

**Figure 2:**
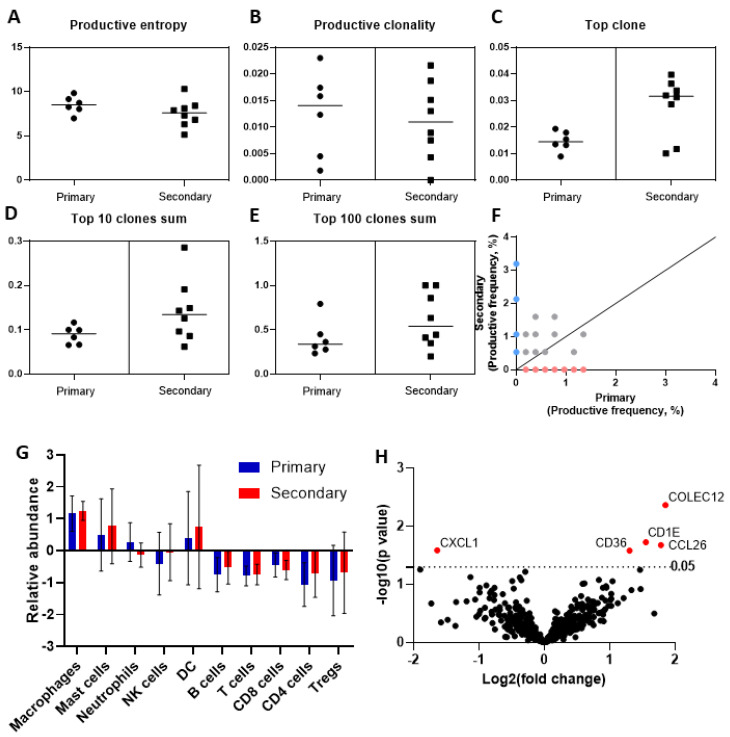
Evaluation of T-cell clonality and transcriptomic heterogeneity in primary and secondary HCC. Measures of T-cell clonality evaluated on the basis of deep sequencing of the T-cell receptor beta chain, using the ImmunoSEQ platform (**A**–**E**). Representative plot illustrating the differences in T-cell clonality, as measured by productive frequency in a representative pair of matched primary and secondary HCC samples (**F**). Targeted transcriptomic profiling, using the NanoString PanCancer Immune profiling panel, highlights homogeneity in the transcriptional signatures reflective of diverse immune cell types across primary and secondary samples (**G**). Volcano plot, illustrating the differentially regulated genes across primary and secondary HCC (**H**).

To assess the heterogeneity of the intra-tumoural immune response across primary and secondary HCC, we performed deep sequencing of the TCR-beta chain, using the ImmunoSEQ platform. Compared to primary sites, the mean frequency of the top T-cell clone was greater in secondary sites (0.028 (SD ± 0.003) versus 0.014 (SD ± 0.01), *p* = 0.016), suggesting expansion of a distinct cell clone in secondary HCC. The mean frequency of the sums of the top 10 (0.088 (SD ± 0.020) versus 0.14 (SD ± 0.07), *p* = 0.09) and top 100 most frequently identified clonotypes (0.40 (SD ± 0.20) versus 0.61 (SD ± 0.30), *p* = 0.18) were not significantly different between primary and secondary HCC, with similar trends observed for productive clonality (mean 0.012 (SD ± 0.008) versus 0.011 (SD ± 0.007), *p* = 0.75), productive entropy (mean 8.4 (SD ± 0.98) versus 7.5 (SD ± 1.54), *p* = 0.20), and other validated metrics of T-cell clonality (Figure 2A–E). T-cell clone productive frequency in a representative pair of samples is shown in Figure 2F.

### 3.5. Phenotypic Differences of the Immune Cell Infiltrate by Targeted Gene Expression Profiling

We complemented the molecular profiling of paired samples by performing targeted transcriptomic analysis of seven matched pairs that satisfied the quality criteria for NanoString analysis. Directed gene set analyses demonstrated no difference in the relative abundance of gene expression signatures belonging to innate and adaptive immune cell types, suggesting equal representation of cell types across sample groups (Figure 2G). Evaluation of individual transcripts revealed significant overexpression of *COLEC12* (*p* = 0.004), *CCL26* (*p* = 0.02), *CD1E* (*p* = 0.02), and *CD36* (*p* = 0.03) and the downregulation of *CXCL1* (*p* = 0.03) in secondary compared to primary samples (Figure 2H,I). To address the possible contribution of the stromal compartment at different secondary sites, we re-analysed the differential expression of these transcripts after grouping by anatomical site (i.e., chest, abdomen) and found that only *COLEC12* was upregulated across sites (abdomen *p* = 0.02, chest *p* = 0.01) (Supplementary Table S1). As only a single sample was available for transcriptional profiling from a liver relapse, this was excluded from re-analysis.

### 3.6. Evaluation of Somatic Mutational Burden in Primary Versus Secondary HCC

**Figure 3:**
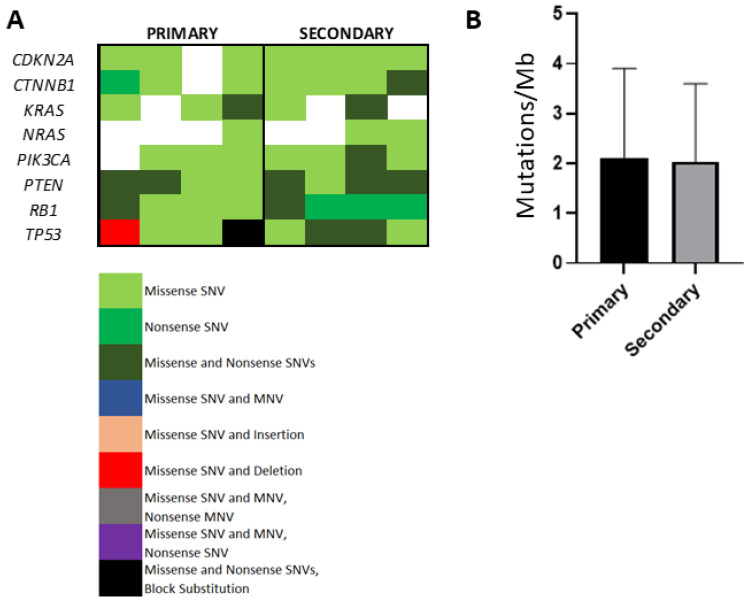
Comparison of the tumour mutational burden (TMB) in primary and secondary HCC lesions. Oncoplot of mutations in recurrently mutated genes in HCC, identified in primary and secondary samples (**A**). TMB estimates in primary and secondary samples, defined as the number of non-synonymous mutations per megabase of assayed genome (**B**).

In view of the influence of higher levels of nonsynonymous somatic mutations in shaping immune tolerance [12], we used tNGS to compare and contrast TMB in primary and secondary HCC. The mean read length was 120 bp (range 90–150 bp) and the total read counts ranged from 80,000 to 455,000 reads/sample. Figure 1A illustrates the distribution of individual somatic mutations across samples, focusing on COSMIC database mutations in genes with previously identified significant levels of mutation in HCC [13]. We recognised, amongst others, the heterogeneous distribution of *TP53* and *CTNNB1* variants across samples. Following adjustment of somatic mutational counts, as described by Buchhalter et al. [14], we reconstructed tNGS-based TMB in primary and secondary samples. There were one primary and two secondary samples that satisfied the coverage-adjusted threshold of 2 Mut/Mb recommended to define TMB-high tumours. We found no difference in the median number of non-synonymous mutations in primary versus secondary HCC (1.7 versus 1.4 mut/Mb, *p* = 0.95, Figure 3B), nor a correlation between TMB and T-cell productive clonality (r = −0.34, *p* = 0.51) or productive reads (r = 0.10, *p* = 0.85).

## 4. Discussion

The immunologic richness of the HCC microenvironment has so far been unhelpful in generating predictive biomarkers for immunotherapy. Whilst transcriptomic studies have highlighted T-cell exhaustion as a prognostic marker in HCC, the strong bias towards the use of resected HCC specimens for biomarker discovery leaves little information as to the similarities and differences of the TME across primary and metastatic HCC. Whether phenotypic characteristics of the T-cell infiltrate are different as a result of a metastatic spread or a recurrence of HCC is a point of greater consequence, as immunotherapy expands across various stages of the disease.

Our study provides a comprehensive and multi-technology assessment of isogeneic primary and secondary HCC samples, combining morphological description with functional characterization of the TME. In our study, we surveyed two cardinal T-cell components of anticancer immunity: the effector, immune-exhausted cytotoxic branch (CD8+/PD-1+) and the T-reg compartment (CD4+/FOXP3+), both of which are linked with poorer prognosis in HCC [15]. Analysis of T-cell spatial distribution revealed poor CD4+ and CD8+ cell infiltration within tumoural areas, where enrichment of CD4+/FOXP3+ was more prevalent. Interestingly, we demonstrated a significant CD4+ and CD8+ gradient across intra- and peri-tumoural areas, suggesting T-cell restriction to the periphery of the tumour. Such spatial disposition, typical of immune-excluded tumours, hallmarks a composite immune-suppressive phenotype, leading to a lack of engagement between effector T-cells and progressive malignancy. Immune-exclusion can be driven by mechanical (i.e., poor vascularity), functional (immune-suppressive crosstalk within the TME) or dynamic barriers (immune-checkpoint receptor/ligand interactions) [16]. In an attempt to elucidate drivers of immune exclusion, we initially evaluated PD-L1/2 expression in primary and secondary HCC and found significant heterogeneity in expression, with evidence of more substantial discordance for PD-L1 tumoural immunolabelling. Site-specific heterogeneity in PD-L1 expression mirrors evidence from lung cancer [17] and combines with analytical heterogeneity as an important factor underscoring the suboptimal performance of PD-L1 in predicting immunotherapy outcomes in HCC. Interestingly, PD-L1+ tumour samples were characterised by denser intra- and peri-tumoural CD8+/PD-1+ T-cell infiltrate, highlighting the central role of PD-L1 as a driver of immune tolerance.

Alongside functional differentiation of the T-cell infiltrate, we looked at somatic mutational burden and T-cell clonality, two inter-related emerging biomarkers of response to ICI. In keeping with previous evidence, we showed that HCC samples are characterised by low TMB across primary and secondary deposits. Deep sequencing of the TCR revealed a largely homogeneous distribution of the TCRB repertoire across primary and secondary tumour samples, using validated measures of T-cell clonality. However, the high sensitivity of our sequencing platform allowed us to identify a significantly different distribution of the top TCRB clonotype across groups, a particularly interesting finding that points towards the existence of a rare T-cell population, enriched in secondary sites. Mapping of the spontaneously occurring expansion of individual T-cell clones is of high translational relevance, given that clonality and its treatment-induced expansion is linked with a response to ICI [18]. Unfortunately, precise detection of the antigen-specificity of the identified TCR is impossible from our data in the absence of paired TCRA sequencing. In addition, the use of archival samples makes it impossible to conduct downstream functional assays, a point that should be explored in future studies.

To overcome these limitations, we performed targeted transcriptomics of tumour samples to complement T-cell phenotyping and clonality data. Whilst supporting the phenotypic homogeneity in the intra-tumoural T-cell infiltrate observed in our immunohistochemistry experiments, our transcriptomic data highlighted differential expression of several genes involved in innate immunity and antigen presentation. A significant limitation of our transcriptional data is that the surrounding non-tumoural compartment is captured, so differential expression limited to a single anatomic site may reflect transcripts in the stroma. Secondary samples showed significant upregulation of *CCL26* and repression of *CXCL1*, suggesting a disturbance of chemoattractants likely to favour myeloid-derived suppressor cells [19]. *CD36*, a scavenger receptor heavily expressed in macrophages, was also over-expressed in secondary samples. Alongside the downregulation of *CXCL1*, this might reflect an immune-regulatory polarisation of macrophages in secondary samples, towards the M2 phenotype [20], although flow cytometry data would be required to validate this. Besides its role in free fatty acid uptake as a scavenger receptor, *CD36* is a positive regulator of angiogenesis, epithelial–mesenchymal transition (EMT) and metastasis in HCC [21]. The relevance of an altered lipid microenvironment in secondary HCC is further supported by the concomitant upregulation of *CD1E*, a non-classical MHC class I-like molecule involved in lipid presentation to T-cells whose overexpression is predictive of adverse prognosis in HCC [22]. Analysis by anatomic site, although limited by small numbers, showed that only one transcript was differentially regulated across anatomic sites—*COLEC12*, a scavenger receptor downstream of TGF-β signalling in EMT-transformed liver sinusoidal endothelial cells [23].

## 5. Conclusions

In conclusion, we present an assessment of the development of the TME across primary and secondary HCC in a unique, albeit small-sized, series of paired tissue samples. We describe preservation of an immune-excluded phenotype between primary and secondary lesions, with no significant differences in the TMB, nor overall T-cell clonality of the immune infiltrate. Transcriptional heterogeneity, related to innate immunity and EMT, contributes to the understanding of the mechanisms underlying immune escape in advanced HCC, and serves as a useful knowledge base for the development of novel therapeutic targets.

## Figures and Tables

**Table 1:** Patient characteristics.

Baseline Characteristics	*N* = 11 [*n* (%)]
Age [median (range)]	69 (44–82)
Year of diagnosis [median (range)]	2006 (1996–2013)
Barcelona Clinic Liver Cancer stage at initial diagnosis	
A	6 (54)
C	5 (46)
Aetiology of chronic liver disease	
Hepatitis C virus	5 (45.5)
Hepatitis B virus	1 (9.1)
Alcohol Excess	4 (36.4)
Cryptogenic	2 (18.2)
Child-Pugh class at first diagnosis	
A	11 (100)
Secondary site (*n* = 13)	
Abdominal wall	1 (7.7)
Cutaneous and subcutaneous	2 (15.4)
Liver relapse	2 (15.4)
Lung	4 (30.8)
Omentum	2 (15.4)
Pleura	2 (15.4)

## Data Availability

Data available from authors upon request.

## Supplementary Materials

The following are available online at https://www.mdpi.com/article/10.3390/cancers13092137/s1, Table S1: Differential expression data (primary/secondary) organised by anatomical sites.

## References

[1] Finn R.S., Zhu A.X. (2021). Evolution of Systemic Therapy for Hepatocellular Carcinoma. Hepatology.

[2] Ang C., Miura J.T., Gamblin T.C., He R., Xiu J., Millis S.Z., Gatalica Z., Reddy S.K., Yee N.S., Abou-Alfa G.K. (2016). Comprehensive multiplatform biomarker analysis of 350 hepatocellular carcinomas identifies potential novel therapeutic options. J. Surg. Oncol..

[3] Swanton C. (2012). Intratumor heterogeneity: Evolution through space and time. Cancer Res..

[4] Stanta G., Bonin S. (2018). Overview on clinical relevance of intra-tumor heterogeneity. Front. Med..

[5] Losic B., Craig A.J., Villacorta-Martin C., Martins-Filho S.N., Akers N., Chen X., Ahsen M.E., Von Felden J., Labgaa I., D’avola D. (2020). Intratumoral heterogeneity and clonal evolution in liver cancer. Nat. Commun..

[6] Kenmochi K., Sugihara S., Kojiro M. (2008). Relationship of histologic grade of hepatocellular carcinoma (HCC) to tumor size, and demonstration of tumor cells of multiple different grades in single small HCC. Liver Int..

[7] Pinato D.J., Vallipuram A., Evans J.S., Wong C., Zhang H., Brown M., Dina R.E., Trivedi P., Akarca A.U., Marafioti T. (2020). Programmed cell death ligands expression drives immune tolerogenesis across the diverse subtypes of neuroendocrine tumours. Neuroendocrinology.

[8] Tsongalis G.J., Peterson J.D., De Abreu F.B., Tunkey C.D., Gallagher T.L., Strausbaugh L.D., Wells W.A., Amos C.I. (2014). Routine use of the Ion Torrent AmpliSeq^TM^ Cancer Hotspot Panel for identification of clinically actionable somatic mutations. Clin. Chem. Lab. Med..

[9] Reuben A., Gittelman R., Gao J., Zhang J., Yusko E.C., Wu C.J., Emerson R., Zhang J., Tipton C., Li J. (2017). TCR repertoire intratumor heterogeneity in localized lung adenocarcinomas: An association with predicted neoantigen heterogeneity and postsurgical recurrence. Cancer Discov..

[10] Monod M.Y., Giudicelli V., Chaume D., Lefranc M.P. (2004). IMGT/JunctionAnalysis: The first tool for the analysis of the immunoglobulin and T cell receptor complex V-J and V-D-J JUNCTIONs. Bioinformatics.

[11] Kirsch I., Vignali M., Robins H. (2015). T-cell receptor profiling in cancer. Mol. Oncol..

[12] Blankenstein T., Coulie P.G., Gilboa E., Jaffee E.M. (2012). The determinants of tumour immunogenicity. Nat. Rev. Cancer.

[13] Ally A., Balasundaram M., Carlsen R., Chuah E., Clarke A., Dhalla N., Holt R.A., Jones S.J.M., Lee D. (2017). Comprehensive and Integrative Genomic Characterization of Hepatocellular Carcinoma. Cell.

[14] Buchhalter I., Rempel E., Endris V., Allgäuer M., Neumann O., Volckmar A.-L., Kirchner M., Leichsenring J., Lier A., Von Winterfeld M. (2019). Size matters: Dissecting key parameters for panel-based tumor mutational burden analysis. Int. J. Cancer.

[15] Tian M.X., Liu W.R., Wang H., Zhou Y.F., Jin L., Jiang X.F., Tao C.Y., Tang Z., Zhou P.Y., Fang Y. (2019). Tissue-infiltrating lymphocytes signature predicts survival in patients with early/intermediate stage hepatocellular carcinoma. BMC Med..

[16] Pai S.I., Cesano A., Marincola F.M. (2020). The Paradox of Cancer Immune Exclusion: Immune Oncology Next Frontier. Cancer Treatment and Research.

[17] Pinato D.J., Shiner R.J., White S.D.T., Black J.R.M., Trivedi P., Stebbing J., Sharma R., Mauri F.A. (2016). Intra-tumoral heterogeneity in the expression of programmed-death (PD) ligands in isogeneic primary and metastatic lung cancer: Implications for immunotherapy. Oncoimmunology.

[18] Tumeh P.C., Harview C.L., Yearley J.H., Shintaku I.P., Taylor E.J.M., Robert L., Chmielowski B., Spasic M., Henry G., Ciobanu V. (2014). PD-1 blockade induces responses by inhibiting adaptive immune resistance. Nature.

[19] Gabrilovich D.I. (2017). Myeloid-derived suppressor cells. Cancer Immunol. Res..

[20] Biswas S.K., Mantovani A. (2010). Macrophage plasticity and interaction with lymphocyte subsets: Cancer as a paradigm. Nat. Immunol..

[21] Nath A., Li I., Roberts L.R., Chan C. (2015). Elevated free fatty acid uptake via CD36 promotes epithelial-mesenchymal transition in hepatocellular carcinoma. Sci. Rep..

[22] Tang X., Shu Z., Zhang W., Cheng L., Yu J., Zhang M., Zheng S. (2019). Clinical significance of the immune cell landscape in hepatocellular carcinoma patients with different degrees of fibrosis. Ann. Transl. Med..

[23] Koudelkova P., Costina V., Weber G., Dooley S., Findeisen P., Winter P., Agarwal R., Schlangen K., Mikulits W. (2017). Transforming growth factor-β drives the transendothelial migration of hepatocellular carcinoma cells. Int. J. Mol. Sci..

