# Supplementary material for: Phenotypic Characteristics of the Tumour Microenvironment in Primary and Secondary Hepatocellular Carcinoma
Source: Cancers (Basel). 2021 Apr 29;13(9):2137. doi: 10.3390/cancers13092137 (PMC8124398; doi:10.3390/cancers13092137)
Supplement: Supplementary file 1 [file cancers-13-02137-s001.zip › cancers-1180466-supplementary.pdf]

**Supplementary Materials**

**Table S1:** Differential expression data (primary/secondary) organised by anatomical sites.

| | **Linear Fold Change in Expression Levels, Primary/Secondary Site (*p*)** | |
|---|---|---|
| | **Abdominal Secondary Sites (Subcutaneous Fat, *n* = 1; Omentum, *n* = 2)** | **Thoracic Secondary Sites (Pleura, *n* = 1; Lung, *n* = 2)** |
| *COLEC12* | **1.6 ($p = 0.02$)** | **9.2 ($p = 0.01$)** |
| *CCL26* | 1.6 ($p = 0.40$) | **15 ($p = 0.03$)** |
| *CD1E* | 1.07 ($p = 0.86$) | **13.7 ($p = 0.01$)** |
| *CD36* | **3.01 ($p = 0.03$)** | 2.87 ($p = 0.16$) |
| *CXCL1* | 0.35 ($p = 0.91$) | **0.14 ($p = 0.04$)** |
